# The Silent Gap : A Clinical Audit of Physical Health Monitoring in a Community Mental Health Team - Plas Gororau, Wrexham CMHT/ BCUHB (Safa Puliyakkadi, Wamiqur Gajdhar, Megan Hughes, Saqib Al Rashiq, Oluseun Abayomi, Asha Dhandapani)

**DOI:** 10.1192/bjo.2026.11700

**Published:** 2026-06-30

**Authors:** Safa Puliyakkadi, Wamiqur Gajdhar, Megan Hughes, Saqib Al Rashiq, Oluseun Abayomi

**Affiliations:** Betsi Cadwaladr University Health Board, Wrexham, United Kingdom

## Abstract

**Aims:**

The objective of this audit is to establish the baseline of how much physical health monitoring is being completed in a general adult outpatient mental health team for patients with severe mental health illnesses (SMI) in accordance with NICE Guidelines.

**Methods:**

Data were collected from CMHT records and the Welsh Clinical Portal for patients who attended outpatient appointments in a CMHT in North Wales over a two-week period in September 2025.

Inclusion criteria:

Patients with a diagnosis that falls under SMI.

Patients prescribed antipsychotics or Lithium.

The Welsh Clinical Portal will be accessed to identify patients who have received a full or partial physical health check from primary care

**Results:**

Out of the 29 patients studied, the following parameters were monitored:

Pulse in 30.4%, Blood Pressure in 56.5%, Weight in 20.7%, Hbs-653C/Glucose in 41.4%, Liver Function in 58.6%, Renal Function in 65.5% and Lipids in 58.6%.

Of the 4 patients who required prolactin testing, 75% had their prolactin monitored.

Comparing patients on Lithium or Clozapine (11 patients) (as they were monitored in dedicated clinics) vs those on other antipsychotics (18 patients), weight was monitored in 45.5% vs 5.6%, Renal Function in 45.5% vs 77.8%, Liver Functions in 36.4% vs 72.2% HbA1c/Glucose in 27.3% vs 50.0% and Lipid Profile 45.5% vs 66.7%.

Comparing High Metabolic Risk Patients (on Olanzapine=5, Quetiapine=7 and Clozapine=3) vs those not on those (14), the monitoring of the parameters was -Weight: 18.8% vs 23.1%, Hbs-653c/Glucose: 50.0% vs 30.8%, Renal function: 75.0% vs 53.8%, Liver Functions: 68.8% vs 46.2% and Lipid Profile: 62.5% vs 53.8%.

Of the patients prescribed Lithium (9/29), Lithium levels were measured ≥4/year in 1/9 (11.1%), Urea and Electrolytes ≥2/year in 6/9 (66.7%), and Thyroid Function ≥2/year in 4/9 (44.4%).

**Conclusion:**

Monitoring of physical health parameters has considerable scope for improvement among patients with SMI in CMHT. Compared with patients prescribed Clozapine or Lithium, those not receiving these medications had their weight monitored more frequently. Interestingly, Renal function, Liver Function, Hbs-653c/Glucose, and Lipids were monitored more closely in the latter group, even though patients receiving lithium and clozapine had dedicated clinics.

The CMHT is planning to allocate a dedicated health worker to monitor all patients with SMI being prescribed antipsychotics. Concordance with the monitoring protocol will be reassessed after this intervention.

